# Poor prognosis of liver transplantation for acute liver failure with acute pancreatitis: Two case reports

**DOI:** 10.1097/MD.0000000000022934

**Published:** 2020-10-23

**Authors:** Liting Yan, Chao Qian, Xin Duan, Jun Ding, Wei Zhang

**Affiliations:** Division of Hepatobiliary and Pancreatic Surgery, Department of Surgery, First Affiliated Hospital, School of Medicine, Zhejiang University, China.

**Keywords:** acute liver failure, acute pancreatitis, liver transplantation, vulnerable state of pancreas

## Abstract

**Induction:**

Liver transplantation (LT) is the only final therapy for patients with acute liver failure (ALF) that cannot be controlled by conservative treatment. Acute pancreatitis (AP) is a recognized complication of ALF. The pathogenesis of AP in ALF patients has not yet been elucidated. The appearance of AP complicates the patients condition and causes a significantly increased risk of mortality.

**Patient concerns:**

We report 2 fatal cases who were both admitted with yellowing of skin and sclera with general weakness lasting for 2 weeks.

**Diagnosis:**

After admission, the laboratory examination of case 1 showed liver dysfunction with serum levels of total bilirubin (TB) 270 μmol/l, alanine aminotransferase (ALT) 106 U/l. Abdominal computed tomography (CT) showed pelvic and peritoneal cavity fluids, occupation of left lateral lobe of liver and unclear margin of pancreas. The clinical laboratory findings of case 2 revealed TB 351.1 μmol/l, ALT 252 U/l, blood lactic acid 18 mmol/l, ammonia 209 μmol/l. And abdominal CT showed pancreatic exudation. They were both diagnosed with acute liver failure, hepatic encephalopathy and AP which was confirmed during the operation.

**Interventions:**

They were both received a routine orthotopic LT.

**Outcomes:**

After the surgery, their liver functions recovered well, and they received conventional conservative treatment for pancreatitis. However, the treatment was not adequately effective, and the infection was too serious and both died of multiple organ failure despite emergency rescue efforts on day 21 and day 19 after LT.

**Conclusion:**

AP is a serious complication that can contribute to prohibitive morbidity and mortality in LT patients. For this reason, the vulnerable state of the pancreas and the scoring system must be defined to help clinicians decide whether a patient is suitable for liver transplantation, and the clinical experience in the treatment of pancreatitis after LT needs to be summarized as an optimal treatment guideline to facilitate better treatment.

## 1 Introduction

Acute pancreatitis (AP) is a recognized complication of acute liver failure (ALF). The mechanism of pancreatitis in patients with ALF remains unknown, but it can be multifactorial. AP in ALF patients is usually asymptomatic and cannot be diagnosed early. Liver transplantation (LT) is the only final therapy for patients with ALF that cannot be controlled by conservative treatment.^[1,2]^ When patients undergo LT, some factors, such as surgical factors, infection, biliary complications and immunosuppression, can aggravate AP processes and finally develop to multiple organ dysfunction syndrome (MODS), causing a significantly increased risk of mortality.^[3]^ Thus, in this condition, the decision to undergo LT and ways to detect, diagnose and treat pancreatitis early should be carefully considered.

In this article, we report 2 fatal cases of patients who developed ALF with hepatic coma and concomitant acute pancreatitis and were treated with LT (Table 1). However, their conditions were too severe, and both died of multiple organ failure on day 21 and day 19 after LT. (Supplemental digital content).

**Table 1:**
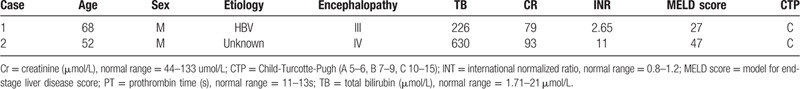
Clinical and laboratory characteristics and scores of the patients before the transplantation.

## 2 Case report

### 2.1 Case 1

**Figure 1:**
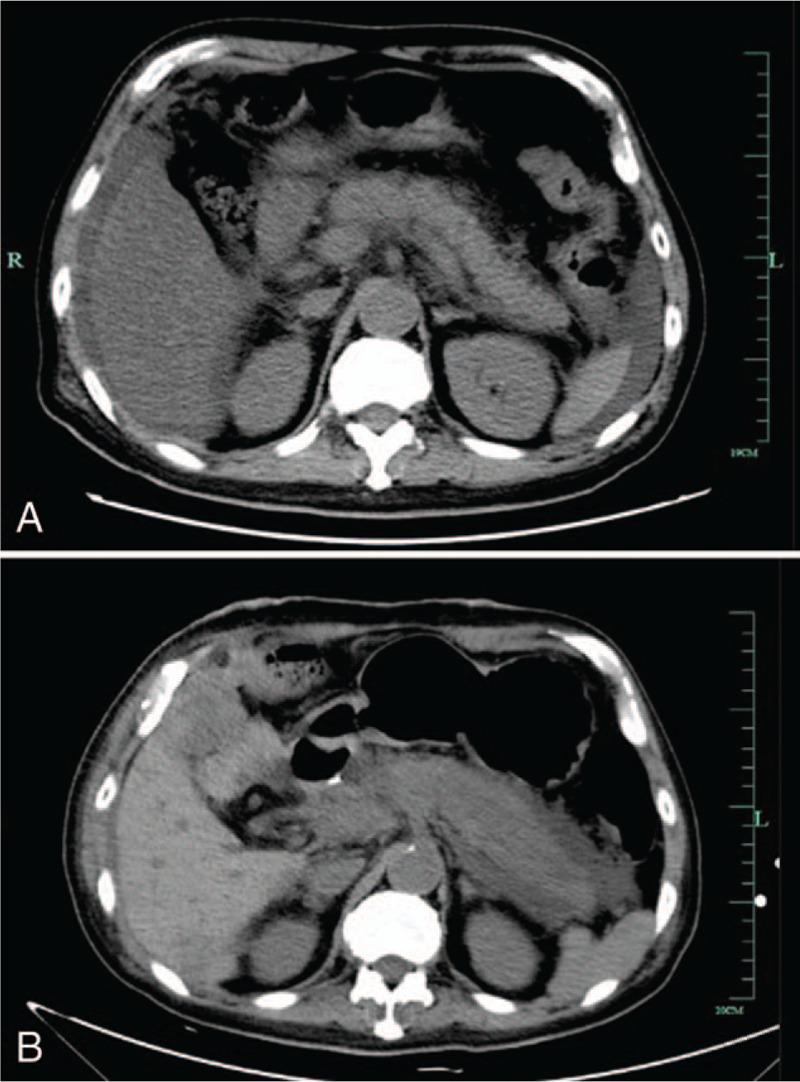
(A) Before surgery, abdominal computed tomography (CT) showed pelvic and peritoneal cavity fluids and an unclear margin of the pancreas. (B) After surgery, abdominal CT showed pancreatic swelling with massive exudation and effusion.

A 68-year-old man was referred to our hospital with a 2-week history of weakness as well as aggravation for 3 days. He had a past history of gastric stromal tumors that were treated with targeted drugs and type-B hepatitis for 40 years, for which no antiviral drugs were taken. On admission, his vital signs were stable with mild yellowing of the skin and sclera. His laboratory examination showed liver dysfunction with a serum total bilirubin (TB) level of 270 μmol/l and alanine aminotransferase (ALT) level of 106 U/l. Abdominal computed tomography (CT) showed pelvic and peritoneal cavity fluids, occupation of the left lateral lobe of the liver and an unclear margin of the pancreas (Fig. 1A). Positron emission tomography–computed tomography (PET-CT) suggested swelling of the pancreas and no recurrence of gastric stromal tumors but metastases in liver. Though he received comprehensive internal treatment combined with artificial liver treatment, his condition deteriorated, and he became unconscious, drowsy and developed hepatic encephalopathy with epigastrium tenderness and rebounding pain over the next few days. He was put on the transplant waiting list with a MELD score of 27 points and received liver transplantation from a deceased donor with an incompatible blood type (AB to A) on day 4 due to his critical condition.

**Figure 2:**
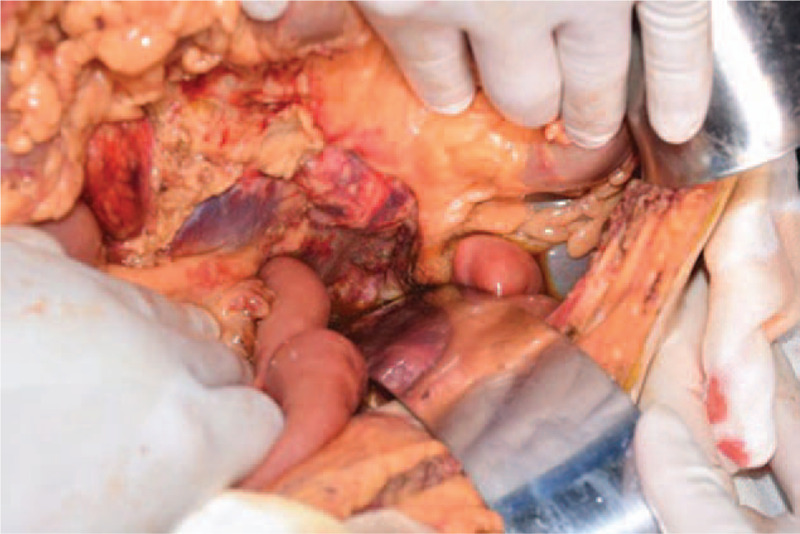
Intraoperatively, hemorrhagic pancreatitis was observed, accompanied by edema of the pancreas.

Intraoperatively, liver atrophy with massive ascites and hemorrhagic pancreatitis were observed, accompanied by edema of the pancreas. The anhepatic time was 100 minutes and the surgery was successful (Fig. 2).

After the transplantation, the patient was sent to the intensive care unit (ICU) for further treatment. His conditions had improved, and he started to use immunosuppressive agents (tacrolimus (FK506) + Mycophenolate mofetil + glucocorticoid) on postoperative day 4. His abdominal CT showed pancreatic swelling with massive exudation and effusion (Fig. 1B), indicating acute pancreatitis on postoperative day 8, while his serum amylase level was normal. His pathogen culture of various body fluids suggested multiple bacterial infections (Klebsiella pneumoniae + Acinetobacter baumannii in ascites culture, candida+ Aspergillus flavus in sputum culture, and Candida parapsilosis in blood culture) though he had received anti-infective therapy after the surgery. Considering his complex condition, he was placed under the care of a multi-disciplinary team (MDT) that decided to place an arrow tube around the liver and tail of the pancreas under B-mode ultrasound guidance instead of operative treatment on postoperative day 11, and coffee-like turbid liquids flowed from the arrow tube. His condition was stable and almost the same as before.

On postoperative day 20, his heart rate suddenly increased, blood pressure decreased, blood gas lactate increased, consciousness was blurred, and light reflex was poor. He received emergency trachea cannula since oxygen saturation could not be maintained, continuous renal replacement therapy (CRRT) for anuria and norepinephrine to maintain blood pressure. On postoperative day 21, he was unconscious, and his internal environment was seriously damaged; he died of septic shock.

### 2.2 Case 2

A 52-year-old man was admitted with a 2-week history of yellowing of the skin and sclera with general weakness. He had a past history of hypertension for 5 years, alcohol consumption and smoking for 20 years. He had no history of viral hepatitis, surgical interventions or drug intake relevant to liver damage. Upon admission, his clinical laboratory findings revealed a TB level of 351.1 μmol/l, an ALT level of 252 U/l, and an ammonia level of 209 μmol/l; his mind was still clear. He was put on the liver transplant waiting list with a MELD score of 47 points under the diagnosis of acute liver failure with hepatic encephalopathy and hepatorenal syndrome. Though he received active conservative treatments, his hepatic encephalopathy developed into a hepatic coma on day 4, and he was admitted to the medical ICU on day 5. In the ICU, he received artificial liver treatment, ventilatory support, continuous renal replacement therapy and norepinephrine to maintain vital sign stability; he also received a routine orthotopic liver transplantation from a non-heart-beating donor with an incompatible blood type (AB to O) on day 6.

Intraoperatively, liver atrophy with little as cites and swelling of the pancreas were observed, confirming acute pancreatitis and the anhepatic time was 52 minutes. The donors liver was suitable for the patient, and the surgery was successful.

**Figure 3:**
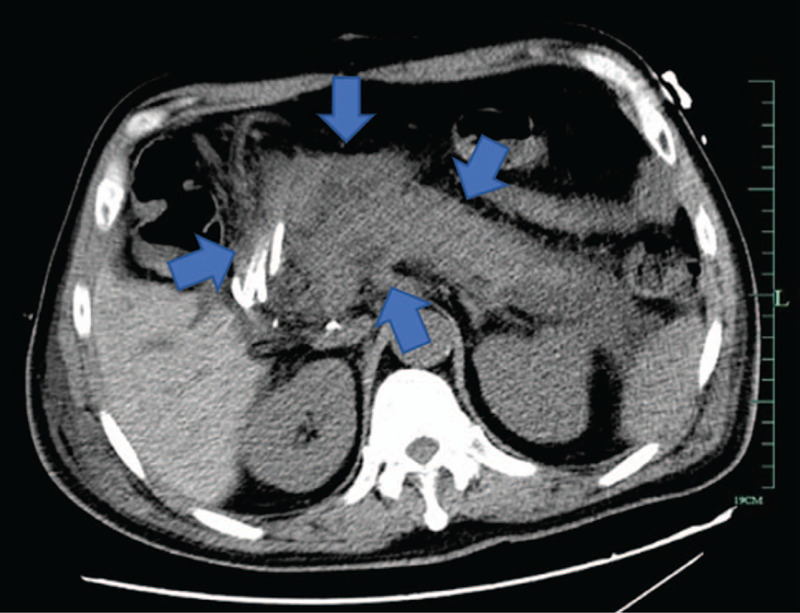
After surgery, abdominal CT showed pancreatic exudation.

The patient regained his consciousness on postoperative day 2, and in the afternoon of that day, his blood pressure dropped; suspecting intraabdominal hemorrhage, he received emergency laparotomy during which surgeons found blood clots around the first hepatic hilum and extensive oozing blood in the surgical wounds. After sufficient hemostasis, the patient was sent back to the ICU and began to revive on postoperative day 3. His condition had improved, and tracheal intubation was removed on postoperative day 7; he started to use immunosuppressive agents (tacrolimus (FK506) + Mycophenolate mofetil + glucocorticoid). On postoperative day 8, the patient showed indifference, a lack of cooperation and his arterial oxygen saturation (SaO_2_) was 0.9 under oxygen therapy; moreover, lung CT showed pulmonary infection and sputum culture suggested Klebsiella pneumoniae. The patient felt chest tightness with a heart rate increased to 150 to 160 times per minute and had abdominal tenderness on postoperative day 11. Abdominal CT (Fig. 3) showed pancreatic exudation, while his serum amylase level was only transiently elevated after the surgery. On operative day 12, he started to have a fever, and his respiratory frequency increased. His blood pressure decreased and was maintained by norepinephrine, while his cardiac troponin (cTnI) was 35.59 ng/ml and B-type natriuretic peptide (BNP) was greater than 5000 pg/ml, suggesting heart failure. Because of the complex situation, the patient was placed under the care of a MDT and was diagnosed by severe pancreatitis with multiple organ damage. Because his condition was too severe and conservative treatment was ineffective, he received exploratory laparotomy on postoperative day 18 that found extensive intraperitoneal pus, pancreatic swelling, and peripancreatic necrosis with hematocele. After debridement and drainage of severe acute pancreatitis, the patient was sent back to the ICU but developed sudden cardiac and respiratory arrest the next day and died despite emergency rescue efforts on postoperative day 19.

## 3 Discussion

ALF is common in China and is usually caused by hepatitis virus, drugs, etc. LT is recognized as the final therapeutic option, while conservative treatment often cannot control the patients condition. In some cases, ALF is accompanied by AP, which can further aggravate the condition, lead to poor prognosis and increase the risk of mortality. Under this circumstance, the role of LT should be taken into careful consideration. There are still no developed guidelines for the specific treatment options for this situation.

These 2 patients both had ALF with hepatic encephalopathy, which complicated the situation.^[4]^ Because of their condition, they were unconscious and unable to express obvious clinical symptoms, though the CT of case 1 showed suspected AP. They were in very bad conditions and almost on the verge of death, so they both received LT with incompatible blood group; during the surgery, AP was confirmed in both cases. And the anhepatic time of these 2 patients was 100 minutes and 52 minutes respectively which had no direct impact on the survival and prognosis of the patients when it was within 100 minutes.^[5]^ After the surgery, their liver functions recovered well, and they received conventional conservative treatment for pancreatitis. However, the treatment was not adequately effective, and the infection was too serious and eventually led to their death.

In adult liver transplantation recipients, the etiologies of AP are not yet clear. Some studies have shown that factors^[6]^ such as a high concentration of free radicals in ALF, an altered coagulation profile, the presence of thrombi, or the hepatitis virus infection itself may result in pancreatic damage and subsequent pancreatitis. Thus, it is urgent to establish the definition of a vulnerable state of the pancreas and a scoring system to help assess this status before surgery in order to help predict the prognosis. However, the specific index of the scoring system needs further studies, such as case-control studies, to identify risk factors including age, sex, CT manifestations, arterial lactate level,^[7]^ high-sensitivity C-reactive protein, etc.; then, these risk factors must be applied in the clinic for proof. When the score of patient gets to a certain value, it indicates that the patients pancreas is in a vulnerable state, which has a high probability of turning into severe pancreatitis after operation. When the patient is in this state, it indicates that the prognosis is very poor, so liver transplantation is not recommended.

Other unavoidable factors can cause or aggravate pancreatic injury, such as intraoperative factors including biliary leakage stimulation, long cold/warm ischemic time, and intraoperative manipulation of the pancreas^[8]^ as well as postoperative factors of using immunosuppressive agents that conflict with infection. These situations require surgeons to operate carefully during the operation to minimize damage to the pancreas. If acute pancreatitis is confirmed during the operation, the early use of somatostatin combined with proton pump inhibitors can effectively reduce body inflammation after the operation.^[9]^ Anti-infection treatment should also be actively administered, and the time and dosage of immunosuppressive agents should be taken into careful consideration to balance infection and anti-immunity to the greatest extent. However, how to effectively monitor the progress of pancreatitis still needs further study.

When conservative treatment fails to control the condition of pancreatitis, we may choose minimally invasive necrosectomy techniques^[10,11]^ such as percutaneous necrosectomy or endoscopic transgastric necrosectomy, which are confirmed to be more safe and effective than open necrosectomy since complications such as large wounds, bleeding and worsening of organ failure are unbearable for patients.

In conclusion, AP is a serious complication that can contribute to prohibitive morbidity and mortality in LT patients. For this reason, the vulnerable state of the pancreas and the scoring system must be defined to help clinicians decide whether a patient is suitable for liver transplantation, and the clinical experience in the treatment of postoperative pancreatitis needs to be summarized as an optimal treatment guideline to facilitate better treatment.

## Author contributions

**Conceptualization:** Liting Yan.

**Data duration:** Liting Yan, Chao Qian.

**Funding acquisition:** Wei Zhang.

**Investigation:** Xin Duan.

**Methodology:** Xin Duan.

**Software:** Jun Ding.

**Supervision:** Xin Duan, Wei Zhang.

**Visualization:** Jun Ding.

**Writing – original draft:** Liting Yan.

**Writing – review & editing:** Jun Ding, Wei Zhang.

## Supplementary Material

Supplemental Digital Content
