# Supplementary material for: Poor prognosis of liver transplantation for acute liver failure with acute pancreatitis: Two case reports
Source: Medicine (Baltimore). 2020 Oct 23;99(43):e22934. doi: 10.1097/MD.0000000000022934 (PMC7581164; doi:10.1097/MD.0000000000022934)
Supplement: Supplemental Digital Content [file medi-99-e22934-s001.docx]

The trend of blood chemistry makers of these two cases from the day of transplantation to the day of death.

CASE 1

CASE 2

ALT, alanine aminotransferase; AST, aspartate aminotransferase; ALP, alkaline phosphatase; GGT, glutamyl transpeptidase; TB, total bilirubin; INR, international normalized ratio; CR, creatinine.
